# Trapped in implicit social exclusion: a study on the restrictive mechanism of male preschool teachers’ identity construction in China

**DOI:** 10.3389/fpsyg.2026.1796491

**Published:** 2026-03-19

**Authors:** Yiling Sun, Nianci Leili

**Affiliations:** 1 School of International Relations and Public Affairs, Shanghai International Studies University, Shanghai, China; 2 Guangxi Academy of Social Sciences, Nanning, China

**Keywords:** androgyny education, identity construction, implicit social exclusion, male preschool teachers, restrictive mechanism

## Abstract

**Background:**

The implementation of the “male-friendly” preschool teacher policy by the Chinese education department for over a decade has marginally increased the proportion of male teachers. However, it is noteworthy that most male students majoring in preschool education have not fully embraced a career in this field as anticipated. Although male preschool teachers are highly valued by preschool education institutions, they continue to exhibit high turnover rates.

**Methods:**

To address the paradox between “high expectations” and “high turnover rates” in the career development of male preschool teachers, we adopted the grounded theory research paradigm to explore the restrictions they encounter during their identity construction process. Through semi-structured interviews with 32 male preschool teachers, we obtained extensive qualitative data and structured the data using NVivo software to present the research findings.

**Results:**

The results indicate that the social environment for men engaging in preschool education has shifted significantly from the direct and overt discrimination of the past. The identity construction of male preschool teachers is now primarily constrained by forms of implicit social exclusion, including cultural exclusion, economic exclusion, and relational exclusion.

**Discussion:**

These exclusions manifest as “unconscious bias” and are embedded within China’s economic, social, and secular cultural structures. They amplify the concerns of male preschool teachers regarding the maintenance of gender norms, and they heighten the pressure and anxiety experienced by men who deviate from traditional gender norms in the process of identity formation.

## Introduction

**Figure 1:**
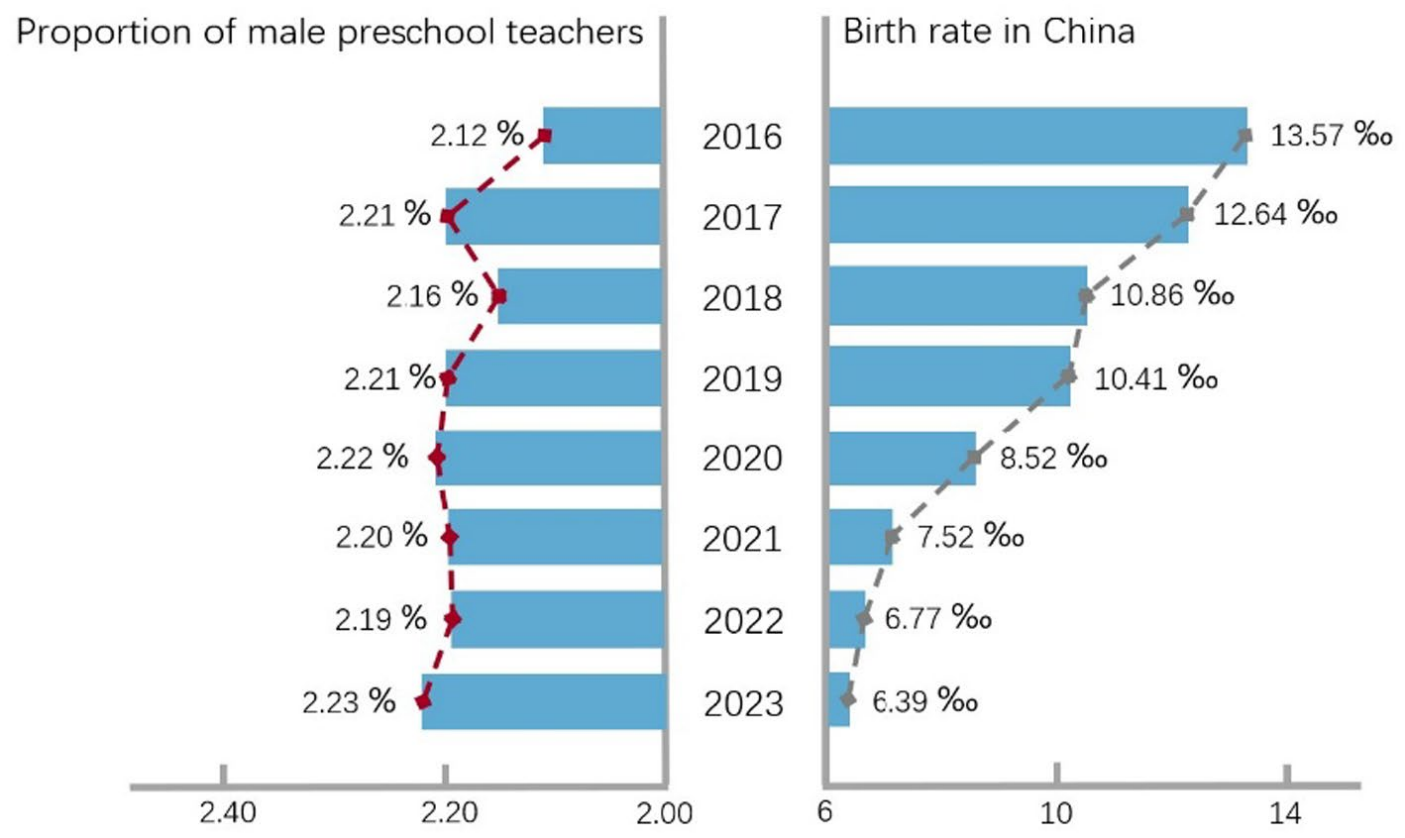
Proportion of male preschool teachers and birth rate in China.

In China, the preschool education system primarily serves children aged 3 to 6, with its core objectives focused on promoting holistic child development and facilitating a smooth transition to primary education. Despite the non-compulsory nature of early childhood education in China, parents widely expect their children to gain a crucial “first-mover advantage” during this stage to ensure adequate preparation for the transition to primary school (Hu et al., 2017; Zhu et al., 2023). Thus, preschool enrollment has become a broadly shared consensus and common practice among households in both urban and rural China. Under the background of pursuing the “first-mover advantage,” the “androgynous education,” which plays an important guiding role in the development of children’s gender awareness, the shaping of personality and the cultivation of social communication ability, has attracted the attention of many parents in China (Hong et al., 2022; Wu and Zhang, 2023). Compared with the traditional “single-sex” preschool education environment dominated by women, male teachers have many “asymmetric advantages” (Gold and Reis, 1982; Sak et al., 2019; Ljunggren and Eidevald, 2023). Consequently, as early as 2010, education departments in certain provinces and cities in China introduced “male-friendly” policies specifically targeting the training of preschool teachers. Through earmarked support and incentives, these policies aimed to attract men to enroll in preschool education programs and focused on increasing the number of male teachers in the field of early childhood education. The goal was to optimize the gender composition of the teaching staff, thereby enabling the concept of “androgynous education” to be advanced within a broader educational perspective (Sun, 2013; Si and Yang, 2022). After more than a decade of implementation, the principles underlying this policy have gradually become known to the public. Through random street interviews conducted in several cities across China, the author found that respondents generally believe the inclusion of male teachers can effectively compensate for the monolithic gender structure of traditional preschools, providing young children with positive and stable gender role models, thereby promoting their more comprehensive and healthy development. These respondents also called for increased recruitment of male teachers in the field of preschool education. This indicates that, guided by the “male-friendly” policy, public opinion in Chinese society regarding men’s participation in early childhood education has become increasingly inclusive. Despite the declining birth rate in China in recent years, the proportion of male preschool teachers has still increased compared to the period before the policy was introduced and has remained stable at approximately 2.2%, without being significantly impacted by fluctuations in the birth rate (MOE of PRC, 2024; see Figure 1).

However, the increase and stabilization in the number of male preschool teachers cannot entirely obscure the underlying issue of high turnover rates. This “male-friendly” policy, though unwritten yet implicitly oriented and structurally supportive, has provided a legitimized pathway for men to enter the field of early childhood education. In practice, however, it has largely failed to convert a substantial number of male graduates from preschool education majors into teachers with stable careers in the field (Yang et al., 2017; Wang J. et al., 2019). Although preschools in China generally welcome the inclusion of male teachers, male preschool teachers still commonly exhibit high turnover rates (Deng, 2017; Feng et al., 2017; Leili et al., 2023). Therefore, this study contemplates why male preschool teachers demonstrate a contradictory high turnover rate despite receiving widespread public support. In the current context where early childhood education concepts are actively shifting toward “androgynous education,” stigmatizing labels such as “pedophile,” “sissy,” or “male nanny” have gradually faded. Nevertheless, when men enter traditionally female-dominated industries, their professional identity construction still encounters a certain degree of subtle exclusion (Derman, 2016). Consequently, this study takes male preschool teachers as its research subjects, employing grounded theory and NVivo software to conduct an in-depth exploration of the subtle social exclusion they face during the process of professional identity construction, trying to explain the high turnover rate of male preschool teachers in China from the perspective of social exclusion.

### Identity of male preschool teachers

Traditionally, early childhood care and education work has been regarded as a “female domain.” In preschools, female teachers not only constitute the overwhelming numerical majority, but their gentle and empathetic gender image is also often considered more aligned with the maternal care and emotional nurturing that young children require (Silman et al., 2019; Haines et al., 2024). Masculinity, by contrast, has been perceived as a symbol of power and a form of gender order standing in opposition to this, with traits such as self-reliance, resilience, and fortitude typically viewed as gender characteristics attached to masculinity (Bourdieu, 2017). Within conventional perceptions and value judgments, these two forms of gender disposition are largely considered incommensurable. Consequently, when men attempt to enter the female-dominated field of preschool education—characterized by “care” and “maternal solicitude”—they are compelled to confront substantial pressure and the challenges of professional stigmatization (Sullivan et al., 2023; Qin and Xiao, 2026). Male teachers in preschool education are thus frequently regarded as having made an “unnatural” career choice and are even labeled with stigmatizing tags such as “homosexual,” “pedophile,” or “effeminate” (Pruit, 2015; Breneselović and Krnjaja, 2016; McDonald et al., 2024). Such negative stigmatization often conveys a derogatory value judgment that preschool education is inherently “women’s work” and that men’s choice to engage in it signifies a “sign of weakness,” thereby depriving them, at the level of gender identity, of the status of being a “real man” (Sargent, 2000; McDonald et al., 2024; Xu, 2025).

In fact, masculinity is a process of continuous construction in practice, which is pluralistic rather than single (Pi and Wang, 2017). In traditional society, the definition of masculinity with an “obsolete sense” ignores the various forms and possibilities of gender practice (Connell, 2003). Contemporary society can no longer simply define the gender traits and behaviors that men should possess through a “sense of gender belonging”. The reconstruction of contemporary masculinity by advanced culture and material civilization has far exceeded the scope of traditional masculinity’s external norms (Schrock and Schwalbe, 2009). However, throughout an extended period of identity construction practices, the connotation and extension of masculinity have neither been fully deconstructed nor have they disappeared compared to earlier stages. Instead, they are still habitually placed within a traditional conceptual framework, regarded as a category distinct from and opposed to femininity (Torre, 2018; Lordan and Pischke, 2022). This implies that men entering the field of preschool education continue to suffer the stigma of “not real men” and are expected to conform to a complete set of traditional gender norms and orders. Correspondingly, existing research often frames the construction of a legitimate professional identity for male preschool teachers as an “inevitable path” in their career development. That is, in their professional practice, male teachers must continuously work to dispel the stigma arising from traditional gender stereotypes and construct a professional identity as a “safe person” who is cautious in physical contact with children and poses no threat of violence (Hedlin and Johansson, 2019; Khamis et al., 2025; Xu, 2025). For instance, in response to skepticism from parents and female teachers, male preschool teachers, while striving to establish their professional advantages, consciously mold their professional identity into that of a “safe person”—one who is cautious in physical contact and exhibits no tendency toward violent threats—in order to mitigate potential societal concerns about men interacting with young children (Bhana et al., 2022; Boadu and Asiama, 2025; Qin and Xiao, 2026). Alternatively, they construct their professional subjectivity between mainstream social norms and gender norms, demonstrating “asymmetric advantages” aligned with gender differences and masculinity, thereby embedding the socially endorsed emotional structure of men into their career development (Xu and Waniganayake, 2018; Yang and McNair, 2021).

With the development of early childhood education concepts, the issue of “male absence” in contemporary preschool education has attracted increasing attention. Consequently, male preschool teachers have come to be regarded as one of the key forces in promoting the diversification of preschool education, and their professional identity construction has accordingly been imbued with numerous expectations (Ljunggren and Eidevald, 2023; Moosa and Bhana, 2023). Many countries have successively transformed their approaches to preschool education development, positioning the attraction of men to enroll in preschool education-related programs and engage in long-term teaching as an important pillar for supporting the diversified and individualized development of preschool education (Jones, 2007; Sullivan et al., 2023). Although the current public discourse environment concerning men engaging in preschool education has significantly improved, with parents of young children and preschool institutions granting greater inclusiveness to male teachers, professional stigma has not entirely disappeared. Instead, it persists in the form of a more subtle “unconscious bias” embedded within socio-cultural contexts, subtly excluding the professional identity construction of male preschool teachers (Derman, 2016). Within China’s highly gendered macro-social structure and traditional cultural background, the value of fulfilling traditional gender role obligations remains deeply entrenched, and male preschool teachers continue to face implicit and persistent pressure from the gender order (Wang X. et al., 2019; Liu, 2019). Previous research on male preschool teachers in China has largely framed the process of professional identity construction as an action continuum that passively responds to the gender order—accepting and acting in accordance with gender role expectations and norms. Such studies presuppose the realistic rationality of responding to “professional stigma” with “justified punishment” to explain high turnover rates. However, in light of current shifts in the public discourse environment, the aforementioned research perspective fails to effectively explain the paradoxical relationship between “high expectations” and “high turnover rates”, overlooking the implicit resistance generated by Chinese society’s gender expectations of men. Therefore, research on male preschool teachers in China needs to further focus on their reinterpretation of professional identity within social contexts, delving deeply into how multi-dimensional exclusion hinders their identity construction.

## Methodology

### Sample

Before the formal interviews, the researcher obtained the contact information of several male preschool teachers through the acquaintance networks of two normal universities. Subsequently, the researcher used snowball sampling to recruit additional potential interviewees and explained the research purpose to them in order to obtain their informed consent. To maximize the representativeness of the sample, the selection of study sites took into account the potential influence of China’s urban development levels on the interview data, thereby avoiding the risk that characteristics specific to a single city might compromise the reliability of the findings. Based on the major cities where potential interviewees were concentrated, purposive sampling was employed to select cities with gradient differences in comprehensive urban social development—specifically a++, a+, and a-type cities (ECUST, 2022). The number of interviewees recruited in each selected city was proportional to the concentration of potential interviewees in that location. The interviewees were selected according to the following criteria:

(1) Initial Screening via Communication: This study selected as potential interviewees male preschool teachers with over 1 year of professional experience who were either currently employed or had formerly been employed in public preschools. Formal establishment positions within China’s public preschool system provide male teachers with multiple safeguards and stable expectations regarding welfare benefits, career development, and professional title evaluation. This sampling strategy effectively mitigates potential threats to the reliability of research conclusions—threats that might otherwise arise from the high turnover rates typically found in private educational institutions due to factors such as market competition, fluctuating human resource demands, and policy changes. During the initial contact and invitation phase, the researcher engaged in informal communication with potential interviewees. This involved explaining the research topic and observing their responses. Candidates who demonstrated a clear understanding of the discussion topic and could articulate their initial thoughts and experiences in a coherent and reflective manner were shortlisted. Conversely, individuals whose communication was predominantly characterized by emotional venting—particularly regarding generalized grievances against their administration or the public-private sector divide—without substantive reflection on their own professional identity, were noted for exclusion.
(2) In-interview Assessment and Final Confirmation: The semi-structured nature of the formal interview itself served as a critical instrument for final assessment and confirmation. Throughout the interview process, the researcher focused on evaluating whether participants demonstrated the capacity to provide detailed and contextualized accounts of their professional experiences, to reflect on the meanings they constructed from these experiences, and to respond constructively to the researcher’s probes and interactions. Participants who consistently offered rich, descriptive information and exhibited metacognitive awareness regarding their career trajectories were regarded as possessing the potential to generate high-quality qualitative data. Interviews that lapsed into repetitive, non-reflective complaints were professionally terminated by the researcher, and the data obtained from them were excluded from the final analysis. This real-time evaluation mechanism ensured that the individuals comprising the final sample functioned not merely as providers of data, but as co-constructors of meaning throughout the research process.

Through purposive sampling, 32 interviewees were selected from Shanghai, Xi’an, and Nanning. From these, 27 interview transcripts (14 from Xi’an, 6 from Shanghai, and 7 from Nanning; comprising 19 currently employed and 8 former teachers) were randomly selected for coding analysis. The remaining five interview materials were reserved for saturation testing.

### Semi-structured interview

Qualitative data were collected through semi-structured interviews. Prior to the formal interviews, the researcher developed an interview guide based on theoretical literature and practical experience. Two interviewees were then selected for a pilot interview, and the guide was revised accordingly. The adjusted interview guide was also sent to the interviewees in advance in written form to allow them adequate preparation. The interview content covered the following topics: motivation for career choice, work content and arrangements, the current work situation of male preschool teachers, and their views on career development. Due to time and geographical constraints, and in order to balance both the quality and efficiency of the interviews, the researcher conducted face-to-face interviews with participants in Xi’an and Nanning during the following periods: September 28 to October 3, 2022; October 4 to October 6, 2022; and February 18 to March 3, as well as April 14 to April 26, 2023. The remaining interviews were conducted via video conferencing software. All interviews took place outside the interviewees’ workplaces, and with their consent, the original audio was recorded to ensure the integrity and accuracy of the interview data. Both interview formats ensured that all participants had sufficient privacy and anonymity to fully describe their professional experiences.

Formal interviews were designed to last between 40 and 60 min. During each interview, while following the main lines of inquiry outlined in the interview guide, the researcher actively encouraged participants to express their views, ideas, and concerns as fully as possible. The researcher also invited them to raise questions and opinions representative of their professional perspective as members of a minority group within the preschool education field. Throughout the interview process, the researcher did not presuppose the research results but kept asking questions closely related to the communication content, actively responded to the experiences shared by the interviewees, and guided them to reflect on the research theme through two-way communication. In order to narrow the distance between the interviewees and make them feel understood and supported, the researchers also flexibly adjusted the interview strategy according to the emotional state and emotional changes of the interviewees.

### Methodological foundation: grounded theory

An individual’s actions are not mechanical responses to external stimuli but are based on the interpretation and construction of situational meanings. Through social interaction, people ascribe meanings to things and adjust their behavior accordingly. Therefore, researchers can delve into the experiential world to understand how actors construct meaning, interpret situations, and navigate the challenges inherent in social interaction (Carter and Fuller, 2016). This epistemological stance influences the methodological orientation of grounded theory (Handberg et al., 2015). Research is not a process of verifying pre-existing hypotheses but rather one of generating theoretical explanations grounded in empirical facts through continuous and iterative data collection and analysis. This methodological approach emphasizes that inquiry should return to the experiential world and build theory from the ground up in a bottom-up manner with an empirical foundation through rigorous coding procedures. Consequently, as a qualitative research methodology, the core tenet of grounded theory is that theory should emerge from systematically collected empirical data rather than being deduced from pre-existing theoretical frameworks (Van and Ukpere, 2014; Charmaz, 2015; Pulla, 2016). Although understandings of grounded theory vary among scholars, its application shares the following core principles: generating theory rather than verifying theory, constant comparative analysis, theoretical sampling, coding and category construction, and the pursuit of theoretical saturation (Eppich et al., 2019; Mohajan and Mohajan, 2023).

The selection of grounded theory as the methodological foundation for this study is based on the following theoretical considerations. First, grounded theory is particularly suitable for exploring research questions concerning “processes” and “interactions.” The central question of this study—why the career development of male preschool teachers manifests a paradox between “high social expectations” and “high turnover rates”—fundamentally involves the continuous interactive process between the individual and society. It also concerns how male preschool teachers perceive, interpret, and respond to various forms of feedback from their social environment within their daily professional practice and social interactions. Second, grounded theory is applicable to the study of phenomena that have not yet been fully theorized. Although existing studies have addressed the professional predicaments of male preschool teachers, few have systematically explained the blocking mechanisms of their identity construction from the perspective of subtle social exclusion. For such research areas where theoretical explanations remain underdeveloped, the methodological advantage of grounded theory—generating theory from interview data—offers unique value. Third, the procedural operational pathway of grounded theory provides rigorous methodological assurance for this study. This research involves the coding and analysis of extensive interview data, necessitating systematic operational procedures to ensure the credibility and traceability of the findings. The three-level coding process of grounded theory—open coding, axial coding, and selective coding—provides a clear analytical pathway, enabling the researcher to engage in continuous dialog between the data and emerging theory, thereby progressively deepening the understanding of the phenomenon (Sancar et al., 2021). Concurrently, the requirement of theoretical saturation testing ensures the empirical adequacy of the research conclusions.

In summary, grounded theory provides systematic methodological support for this study. By adhering to the research procedures of grounded theory, the empirical grounding and theoretical depth of the research findings are ensured. This enables the study to offer original theoretical explanations for understanding the phenomenon of high turnover rates among male preschool teachers in China.

### Coding process

Qualitative data provide a rich empirical foundation for this study, facilitating an in-depth understanding of the internal logic underlying the actions of male preschool teachers and enabling the tracking and analysis of how situational factors influence their behavior. To obtain well-substantiated qualitative findings, this study employed NVivo software to structure the large volume of interview data. The data processing followed the procedures of grounded theory and was conducted in accordance with the three-step coding paradigm of “open coding - axial coding - selective coding”. Throughout the coding process, continuous theoretical dialog was maintained with the empirical material, accompanied by a sustained reflective and critical awareness.

(1) Open coding. Open coding emphasizes setting aside traditional theoretical presuppositions and analyzing all potential meanings embedded in the original materials through a brainstorming approach. It seeks to explore the essence of the ideas expressed in the data and to assign appropriate conceptual categories to these ideas (Strauss and Corbin, 2015). The coding process involves integrating similar raw concepts into initial categories by extracting meaningful keywords, sentences, paragraphs, and other units of analysis.
(2) Concepts with a coding frequency of two or fewer were either eliminated or revised in this study. In addition, certain lengthy sentences that were difficult to disaggregate were occasionally classified into two or more initial categories simultaneously. Accordingly, when encoding such textual data, these sentences needed to be coded concurrently under different initial categories.
(3) Axial coding. The process of axial coding involves exploring the potential logical relationships among the initial categories and grouping similar categories based on their attributes (Marshall and Rossman, 2014). The primary purpose of axial coding is to analyze and compare the discrete initial categories formed in the open coding stage, and connect them into major categories with internal logic according to different conceptual dimensions, so as to make the causes and context of the problem well-founded.
(4) Selective coding. Selective coding aims to explore the correlations and commonalities among the major conceptual categories identified during axial coding, and to form a core category that can explain the entirety of the research problem (Jia and Heng, 2020). The task at this stage is to establish a central node that integrates all major categories and clearly articulates the “story theme.”
(5) Theoretical saturation test. In order to test whether the theoretical construction tends to be perfect, the theoretical saturation is tested with the remaining 5 interview materials. After analyzing 5 interview materials according to the above coding method, no new concepts and categories are found in the results. Therefore, it can be concluded that the study has reached theoretical saturation and demonstrates sufficient explanatory power regarding the phenomenon under investigation.

### Data presentation

According to the coding rules and grounded theory, the original concepts could be integrated into nine initial categories, such as “weak purchasing power of necessities” and “mainstream gender discourse pressure” on the basis of open coding. It could be observed from the initial categories that the experiences of male preschool teachers mainly involve their perception of subtle, vague, and sometimes unintentional hints in social situations, which typically carry negative connotations. The micro-erosion of individual emotions by these negative experiences is precisely the remarkable feature of social exclusion, suggesting that the individuals are labeled as “inconsistent” (Baumeister and Leary, 1995; Bastian and Haslam, 2010). Referring to the classification of the main types of social exclusion (Eck and Riva, 2016), the 9 initial categories can be abstractly integrated into three major categories: “economic exclusion,” “cultural exclusion” and “unconscious relationship exclusion.”

The major categories showed that the process of identity construction of male preschool teachers is closely related to social interaction. In fact, identity consists of objective identity and subjective identity, which includes not only the social discipline on individual action goals, behavior patterns, compliance with norms and role obligations, but also the individual’s presentation of identity characteristics, construction of identity significance and resonance of emotional experience brought by identity (Tajfel and Turner, 2004; Liu, 2014). It can be seen that identity is based on the interaction between individuals and society, and ultimately depends on self-perception, recognition and acceptance of identity. Therefore, the perception of social exclusion directly affects the construction of self-identity, and makes identity have both social attributes and personality consciousness. Finally, the “story theme” of this study could be summarized as “low self-identity: the rupture between identity and social acceptance” (see Table 1).

**Table 1:** The coding of interview materials.

Core category	Major category	Initial category	Original concepts (examples)
Low self-identity: the rupture between identity and social acceptance	Economic exclusion	A relatively low welfare treatment	… I heard that the salaries and bonuses in preschools are lower than those in primary schools (N12)
		Weak purchasing power of “necessities”	Compared with my classmates in other occupations, my salary is not enough to pay the mortgage (N5)
		The ability to expand additional income	It is almost impossible for male teachers to make use of professional skills to earn more income (N17)
	Cultural exclusion	The pressure of mainstream gender discourse	By the age of 30, I am definitely not a mature and steady person in the traditional social concept … (N19)
		Contrary between professional image and social image	My girlfriend asked me, how am I going to teach kids to dance when I’m 50? (N8)
		Amplification and misinterpretation of media traffic	… Some questions about malicious hype of male teachers from the media are just to attract traffic (N1)
	Unconscious relationship exclusion	An ambiguous teacher’s identity	… and they think I’m a male nanny rather than a real teacher (N7)
		De-embedding of work and life	Most of my friends work in enterprises. From chatting, I can feel that they are not interested in my work (N14)
		non-malicious teasing in social situations	… Everyone asked me to show how to teach children to dance in preschool, which made me feel embarrassed (N23)

After data collection, a total of 907 coding contents are sorted out. The coding structures of the main categories are “economic exclusion” (46.7%), “cultural exclusion” (41.2%), and “unconscious relationship exclusion” (12.1%) in turn. Among them, there are 423 codes related to “economic exclusion,” which is consistent with the conclusion that salary has a large proportion on career stability in previous studies, indicating that welfare treatment is still a key factor affecting preschool teachers’ career choice. Male preschool teachers have low satisfaction with the current salary, and the market rule of “a thing is valued if it is rare” has not happened in their work practice. Salary and future development trend are still the basic issues that male preschool teachers are most concerned about. The content of “cultural exclusion” totaled 374, and the high-frequency keywords of NVivo such as “responsibility,” “status,” “success” and “conscientiousness” reflected the potential pressure exerted by traditional society on male preschool teachers to realize their economic aspirations and social status to some extent. It shows that within the socio-cultural context of contemporary China, men are still expected to fulfill a complete set of traditional gender norms and face the anxiety of “establishing a family and career on schedule.”

The smallest number of codes was for “unconscious relationship exclusion,” at 110. However, it is worth noting that from the process of coding, it can be found that the generation of “relationship exclusion” is based on economic exclusion and cultural exclusion—when male preschool teachers participate in social activities of peer groups, their friends are affected by the economic, cultural and professional concepts of traditional society, and fail to give adequate understanding and recognition to the significance and value of their work. Some teasing and unconscious prejudice on their work lead to the marginalization of male preschool teachers’ interpersonal relationships in peer groups, and even lead them to avoid or not participate in social occasions.

## Explanatory framework

### Attribution analysis of restricting identity

In fact, the social environment plays a key role in the process of constructing individual identity, and social culture, values and interactive feedback with peer groups and relatives will have an impact on individual identity (Stewart and Brown, 2019). These obscure and implicit forms of social exclusion are responsible for disciplining and guiding men to “play” gender roles, fulfill gender expectations, and assume gender obligations. Individuals often strive to meet social expectations and gender expectations, but at the same time they are bound by these expectations, leading to contradictions and troubles in personal self-identity. Therefore, it is necessary to further understand the types and concrete manifestations of social exclusion that hinder the construction of male preschool teachers’ identity.

#### Cultural exclusion—“how am I going to teach kids to dance when I’m 50?”

The narrative logic underlying the transition from traditional society to modern society—characterized by rapid economic growth and urbanization—often points to an increasingly significant social issue: the erosion of traditional culture by increasingly materialized lifestyles. In contrast, although China’s modernization process has profoundly shaped contemporary life, its local culture remains deeply embedded with traditional value systems. These include discursive elements such as “authority,” “status,” “self-respect,” and “achieving success and winning recognition”, which impose implicit expectations on male role performance that diverge considerably from “modern discourse” (Song, 2018; Wei, 2026). Influenced by traditional cultural norms that emphasize gender role obligations, young people in China still tend to psychologically associate gender responsibilities with traditional cultural expectations. Consequently, men navigating the space between traditional and modern cultural frameworks often exhibit a strong traditional orientation in their social participation. They perceive themselves as morally obligated to prioritize societal gender role expectations and regard the fulfillment of these obligations as a crucial foundation and means of self-realization (Yang, 1998; Hu et al., 2018). When men enter the professional field as preschool teachers, they encounter a fundamental tension—their professional role conflicts with the traditional male image and societal expectations in China. A pressing issue they face upon entering the profession is that traditional society implicitly expects them to assume the responsibility of shaping and maintaining a conventional masculine image, as well as achieving secular career success. Career success, in this context, encompasses multiple dimensions—most notably, gaining social status, securing professional discourse power, and embodying both a masculine image and economic conditions aligned with conventional gender stereotypes. Therefore, with the ongoing accumulation of gender-related social experiences and the progression of age, male preschool teachers develop an increasingly pronounced need for an image of maturity and stability, as well as for professional success (Yang and Delores, 2019).

My girlfriend asked me, how am I going to teach kids to dance when I’m 50? Therefore, she asked me to be a public primary school teacher or a civil servant… Will I become a preschool leader in the future? I have no idea. If I can’t become a leader, then as I get older, I may find it unbearable to remain in the same work situation. (N8)

In their professional practice, male preschool teachers must cultivate a “playful big kid” image to integrate into the early childhood education environment and win children’s favor, as required by pedagogical needs. During interviews, most male teachers reported that when contemplating their long-term career plans, they realize the professional image prescribed by the preschool education field stands in stark contrast to traditional templates of masculinity. In the workplace, they must constantly maintain the demeanor of a “tough guy with a tender heart,” striving to present themselves as approachable and trustworthy “older playmates.” Outside the workplace, male preschool teachers must constantly cope with pressure from relatives, friends, and girlfriends or wives, stemming from the secular discourse that traditional men should be steady and achieve both success and fame.

… If I have been engaged in preschool education for a long time… I can’t imagine what’s going to happen. But I think when I get to a certain age, 40 or 50, if I’m still playing with kids in preschool, I don’t think I can bear it, let alone my family. (N20)

Within the context of traditional culture, the pursuit of a male image that conforms to mainstream gender norms remains widespread. Discursive and social constraints lead young men to unconsciously accept and identify with male role obligations prescribed by traditional culture, thereby bearing certain gender and social expectations during the process of re-socialization (Liu and Ding, 2022). As age increases, the tension between the “playful image” of preschool teachers and the “mature and steady” image of traditional men gradually intensifies, placing male preschool teachers under considerable pressure to integrate their gender images in real life. Confronted with the divergence between their professional image and the traditional social image, male preschool teachers must constantly negotiate the image gap inside and outside the workplace, integrating their identity and gender roles into a personal image that is persuasive both to themselves and to others. For these teachers, the capacity to cope with the pressure of image integration diminishes with age, gradually leading to pronounced integration difficulties.

#### Economic exclusion—“am I a man who lives off a woman?”

With economic and social development, the social status of women in China has significantly improved, and their educational attainment has gradually caught up with that of men (Treiman, 2013; Wu et al., 2014). In fact, men and women in China generally possess comparable employability and equal rights to participate in economic and social development. However, in private life, young men and women in China still adhere to quite traditional gender concepts (Yan, 2002). It is widely believed that high income, a well-dressed appearance, and material wealth represent the essence of masculinity. Particularly regarding the division of family responsibilities, men who assume a greater share of material and economic provision than their partners are often regarded as more masculine (Gerlitz, 2023; Ishizuka, 2025). For male preschool teachers, although the “male-friendly” policy facilitates their entry into the profession, the decision to leave is fundamentally intertwined with tangible economic barriers, particularly salary levels. In China, the early childhood teaching profession has long been characterized by a discrepancy between high societal expectations and relatively low financial remuneration. Although male teachers are often welcomed for their perceived unique contributions, their compensation remains disproportionately low compared to many other occupations requiring similar educational qualifications (Yu, 2025). This economic reality constitutes not merely a contextual factor, but a direct obstacle to job retention.

On the one hand, as consumerism occupies the mainstream cultural ideology of contemporary Chinese society, economic conditions are becoming increasingly important as a determinant of male value (Alpermann, 2012). Since the transition to a market economy, people in China have generally regarded economic conditions and economic potential as key indicators for evaluating men’s worth and charm (Zurndorfer, 2016). Under the influence of commercialization and consumerism, high-end consumer goods—such as homes in school catchment areas, luxury cars, and designer products—are idealized as “necessities” for a healthy life and family happiness, placing immense financial pressure on young men who have just entered the workforce. In an increasingly competitive and money-oriented society, a man’s lack of such “necessities” often signifies a loss of social status and prestige, and may lead to feelings of inferiority within social networks (Osburg, 2013). On the other hand, strong economic conditions and economic potential constitute the power base for traditional Chinese men to maintain a dominant position in gender relations (Liu and Ding, 2022). In contemporary gender ideology and various manifestations of gender inequality, the most salient determinants are often closely associated with the disparity in economic power between the sexes (Louie, 2014). Although most of the media and TV dramas frequently portray contemporary Chinese couples as mutually respectful and supportive, beneath the rise in women’s economic status lies an underlying male anxiety over the potential loss of dominance in gender or family relations (Zurndorfer, 2016). Consequently, in managing gender relations, men tend to actively embrace the traditional role of “breadwinner,” demonstrating their masculinity respectably by shouldering a greater share of economic expenses.

… My girlfriend works in an insurance company, and her salary is higher. For the convenience of going to work, she bought an electric car. She takes me to the preschool first in the morning, and then she drives to the company. In recent years, her salary has indeed increased much faster than mine. My monthly income does not change much. Am I a man who lives off a woman? Ha-ha… It’s a little awkward… (N5)

In the view of respondent “N5,” being economically inferior to his partner is regarded as “undignified” and “embarrassing,” as this situation challenges the traditional expectation that men should occupy the dominant economic position within intimate relationships. However, the relatively low salary of preschool teachers has long been a persistent issue in China’s early childhood education sector (Liu and Zeng, 2021). Adequate compensation and benefits are associated with stronger career commitment and lower job burnout. Although male preschool teachers’ initial motivations for entering the profession—such as future aspirations, professional curiosity, employment pressure, or a sense of dedication—were not primarily driven by economic considerations, they gradually come to realize, amid the intensifying “struggle between roses and bread”, that traditional society continues to place significant value on individuals’ “pragmatic” economic potential and conditions. During interviews, most male preschool teachers acknowledged their strong preference for urban life and recognized that positions within the public system provide relatively robust social security and stable working conditions. Nevertheless, when compared with primary school teachers, secondary school teachers, or professionals in other industries, the workload in early childhood education remains disproportionate to income. Against the backdrop of relatively modest wages in the preschool sector, the future economic burdens faced by male preschool teachers—including marriage and dating expenses, childcare costs, leisure consumption, and the maintenance of interpersonal relationships—are more likely to constitute multidimensional economic exclusion in their daily lives (Bullough et al., 2014). Confronted with slow personal income growth, limited prospects for welfare improvement, and socially established “necessities” for marriage and family formation in China—such as housing, cars, and betrothal gifts—the economic pressure on young male preschool teachers to fulfill the traditional role of “breadwinner” has intensified markedly.

#### Unconscious relationship exclusion—“everyone asked me to show how to teach children to dance in preschool”

The presentation of people’s social attributes largely depends on information exchange and interaction within social groups. Therefore, developing and maintaining interpersonal relationships has become a key component of most people’s daily lives (Wyer and Schenke, 2016). Like others, male preschool teachers also seek to gain broader understanding and recognition of their profession through interpersonal communication. However, because traditional gender discourse and mindsets have long dominated societal understandings of masculinity, instances of male preschool teachers being implicitly excluded by “others” in social interactions and interpersonal networks remain relatively common (Pruit, 2014).

Unlike the relational exclusion experienced by marginalized groups such as the impoverished or chronically ill—which often manifests as outright refusal of contact, social distancing, or even overt hostility—the exclusion reported by male preschool teachers in this study predominantly occurs within the context of everyday interactions with others. In their professional practice, the career training and development pathways provided by preschools for male teachers are rarely gender-neutral; more accurately, they are typically modeled on those designed for female teachers. For instance, training content for male preschool teachers tends to emphasize nurturing care, emotional sensitivity, fine motor skill activities, and classroom management techniques—attributes aligned with traditional female pedagogical archetypes. While these are foundational skills for early childhood educators, our interviews reveal that male teachers often feel frustrated by the lack of attention to and utilization of their so-called “asymmetric advantages” in professional practice. Opportunities for male teachers to showcase their unique strengths—such as guiding children in more physically active play or fostering children’s risk-taking abilities—remain limited. This undifferentiated approach to professional training and development inadvertently overlooks the distinct professional identity many male teachers seek to construct, making it difficult for them to establish professionally valuable contributions that reflect gender differences within the female-dominated early childhood education field. Implicitly, this exacerbates their sense of marginalization in team interactions and fosters subtle relational distance with female colleagues—who often perceive male teachers as mere “tokens” whose presence fails to transcend biological difference in any substantively meaningful way. Beyond the workplace, the sense of dissonance male preschool teachers experience regarding traditional gender images and economic status is primarily evoked through unconscious teasing in social interactions and a lack of recognition of their “value as teachers”.

Once at a party, we talked about a video of Tik Tok, in which an internet celebrity teacher led the children to perform a children’s dance. Everyone asked me to show how to teach children to dance in preschool, which made me feel very embarrassed… (N23)

A friend was laid off and complained about the recent bad employment situation. I suggested that if he could not find a job with good welfare benefits for the time being, he could find a simple job first and get at least some income every month. He thought I wanted to recommend him to work in preschool, and replied that he would not waste his youth in preschool with children … I think they subconsciously feel that my work is worthless, but they just didn't tell me in person. (N17)

Although the male preschool teachers in this study were not directly or actively excluded from their peer networks solely for engaging in a “feminine profession,” this does not imply that they receive full recognition or acceptance within social interactions. In fact, through their sustained awareness of “stereotype threat” in everyday contexts, these teachers gradually become conscious of a significant incongruity between themselves and the masculinity norms unconsciously upheld by their peer groups. This incongruity does not stem from overt behavioral conflict or value opposition, but rather resides in seemingly trivial yet symbolically charged details embedded in daily interactions. In the interpersonal encounters of male preschool teachers, this threat often manifests subtly in the form of “non-malicious teasing” or “unconscious bias.” For instance, during social gatherings, a male teacher may be playfully asked to “perform a preschool dance”, or, when career choices are discussed, be jokingly questioned whether he “entered early childhood education to escape societal pressures”. Although such remarks may appear lighthearted, unintentional, or even affectionate, they implicitly convey the assumption that engaging in preschool teaching is an “atypical” pursuit for men—suggesting that men are expected to choose professions perceived as more authoritative, technical, or financially rewarding, while early childhood education is regarded as an exceptional path incongruent with conventional masculinity. Despite the absence of explicit exclusionary intent behind such teasing or bias, for the male teachers on the receiving end, these interactions transmit a subtle yet unmistakable signal: their professional identity has not achieved genuine normalization within their social circles. Individuals are typically highly sensitive to the implicit devaluation or rejection embedded in such unconscious exclusion, which can evoke psychological distress, feelings of inferiority, and gradually lead to the internalization of external judgments, fostering self-doubt regarding their self-worth (Kerr and Levine, 2008). Consequently, in the process of maintaining interpersonal relationships, male preschool teachers find it difficult to attain authentic affirmation of their profession and identity through social interaction. They are confronted with a social predicament characterized by being “neither fully expelled yet perpetually peripheral”. This situation renders “stereotype threat” not merely an episodic psychological burden, but an enduring and significant source of masculine role pressure. It compels them to continuously navigate the implicit disciplinary effects of traditional gender norms within their social lives, ultimately precipitating confusion and anxiety in their self-identity formation.

### Restriction mechanism of identity construction

**Figure 2:**
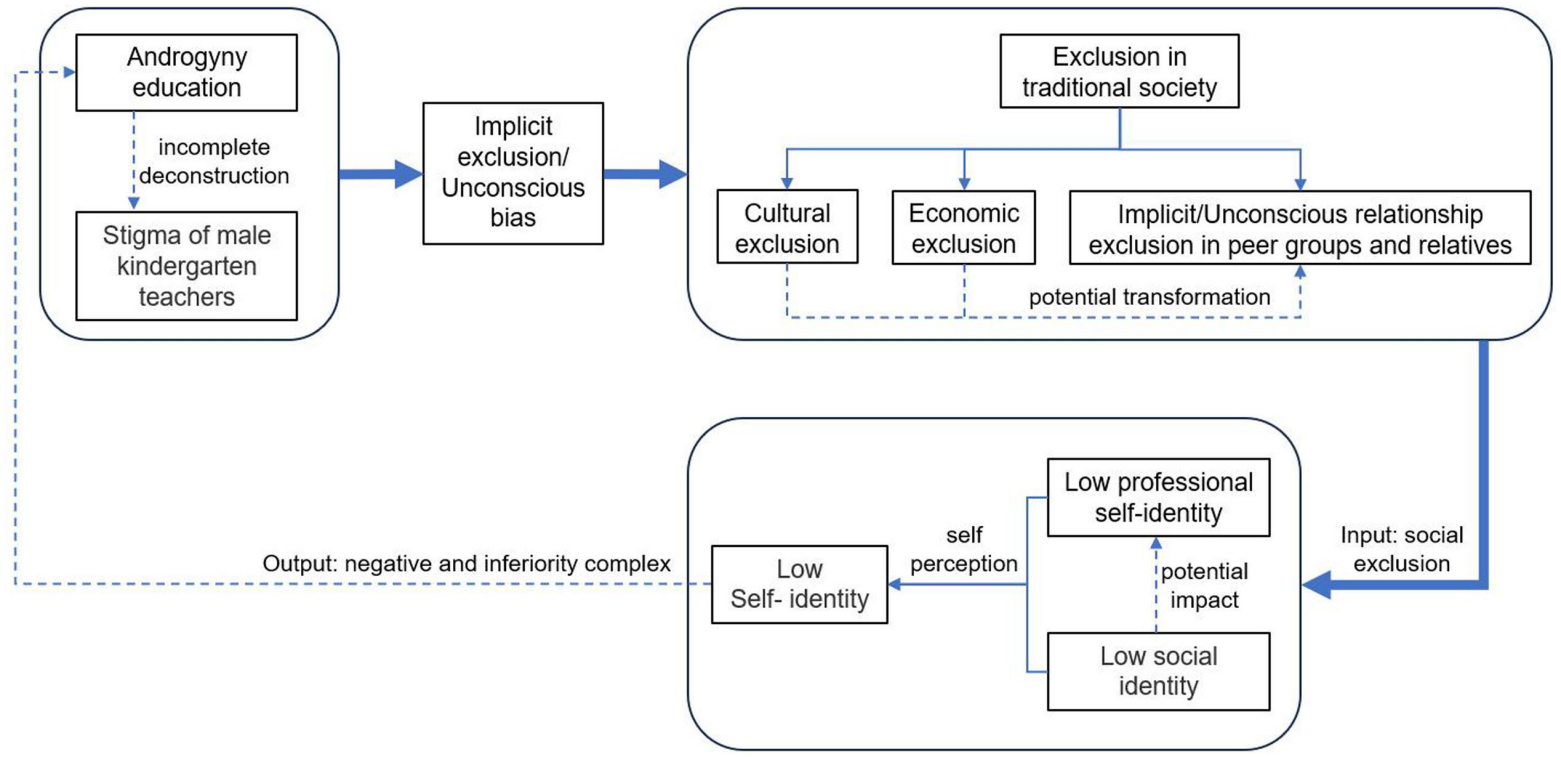
Explanatory framework of restriction in identity construction.

The long-term publicity of the “male-friendly” policy has created a strong “halo effect” for men entering the non-traditional profession of preschool education. Interviews revealed that, based on trust in the policy and expectations regarding the application of professional skills in early childhood education, men aspiring to pursue this career generally possess strong confidence in overcoming workplace prejudice and anticipate fully leveraging their asymmetric advantages in professional practice. However, the role of the “male-friendly” policy largely ends at attracting men into the field. Once recruited, male preschool teachers’ identity construction remains constrained by traditional gender norms (Leili et al., 2023). Although the growing emphasis on “androgynous education” generates substantial practical demand for male teachers, and despite their being welcomed by many preschool institutions, male preschool teachers remain a “minority group” in reality. The accumulating tension between their professional image and the traditional gender order gradually exposes them to implicit and unconscious forms of social exclusion (see Figure 2).

The development of the market economy since the reform and opening-up has profoundly influenced China’s economic and social structure, while also gradually aligning the social image of Chinese men with global norms through increased cross-cultural contact and mutual understanding. However, this alignment does not signify a simple rupture between Chinese society and traditional gender ideology. On the contrary, certain customs and gender perceptions remain deeply embedded in everyday gender practices, reflecting a profound inertia in traditional values concerning gender order and family responsibilities (Yan, 2002; Hu et al., 2018). Within traditional Chinese culture, prevailing views on masculine characteristics hold that a man should first and foremost embody a male image consistent with the established gender order. This image may take the form of a “gentleman” in intellectual pursuits or a “rugged man” in physical labor, but it must never appear weak, effeminate, or immature (Zheng, 2015). Through cultural discipline, men unconsciously internalize and align themselves with traditional gender discourse, shaping and remodeling their gender performance according to an implicit “obligation to enact masculinity” (Liu and Ding, 2022). This process is particularly pronounced in the competitive environment of China’s contemporary market economy, where the construction of gender image is often contingent upon the demonstration of ambition and potential as reflected in one’s economic wealth, social resources, and career aspirations (Zurndorfer, 2016). Furthermore, influenced by traditional family values, Chinese society generally presumes that men bear primary responsibility for breadwinning. Consequently, the ideal male image is portrayed as one capable of providing substantial economic or material support for shared goals within intimate relationships—such as purchasing a home, a car, or funding travel. Although instances in which women out-earn their male partners have long been common in China, the anxiety associated shouldering greater breadwinning responsibilities continues to be interpreted as evidence of men fulfilling their gender obligations (Osburg, 2013). Thus, within peer relations, the valorization of traditional masculine ideals amplifies attention to the performance of gender duties, imposing greater pressure and anxiety on men whose identities deviate from conventional gender norms as they navigate the process of identity construction.

In their gender practices, male preschool teachers commonly perceive that society subtly conveys to them multiple, intersecting pressures related to social status, professional image, and economic income. These pressures indicate that traditional gender discourse and stereotypes continue to dominate societal understandings of masculinity, requiring male preschool teachers to conform to gender norms prescribed by mainstream social culture. Although male teachers generally believe that the “playful big kid” persona they project aligns with professional expectations, this “minority group” often fails to receive objective recognition from mainstream gender discourse when attempting to integrate their professional identity into social life. Consequently, male preschool teachers must continually navigate the tension between their “playful big kid” professional image and the socially expected image of being “mature and steady” across various life contexts—such as social interactions and marriage—in an effort to garner broader understanding and recognition of their profession. However, as they age and their economic burdens intensify, the long-standing and irreconcilable contradictions involved in integrating their professional and gender identities, as well as navigating self-expectations versus societal expectations, progressively erode the subjective identification male preschool teachers have constructed around their professional values, beliefs, and emotions. This erosion gives rise to thoughts of changing careers while still young, manifesting as increasingly fragile professional commitment. Consequently, it emerged from interviews with the majority of male preschool teachers that the acute sense of urgency and immense pressure conveyed by implicit social exclusion lead them to perceive preschool teaching not as a “long-term meal ticket”. Instead, a common practice has emerged: “keeping one’s options open” by obtaining alternative certifications or preparing for civil service examinations.

### Recommendations

The “male-friendly” preschool policy has contributed to a more balanced gender composition within China’s early childhood education workforce, achieving a certain degree of success in attracting men to the profession and promoting the concept of androgynous education. However, the interviews revealed a lack of career development pathways specifically tailored for male teachers within public preschools. Consequently, from the perspective of professional role construction, while the policy has facilitated men’s entry into the field, it fails to address the distinct needs of male preschool teachers in their career progression, nor does it mitigate the retention challenges arising from implicit social exclusion. To effectively reduce the high turnover rate and retain male teachers in preschools, more robust policy support and targeted guidance for career development remain imperative.

(1) Translating gender advantages into professional competence. Within the current context of preschool education oriented toward androgynous education, society expects male preschool teachers not only to keep pace with professional advancements but also to demonstrate and leverage the distinctive advantages associated with their gender. Preschool administrators should therefore recognize the intrinsic connection between gender strengths and professional practice, and actively create favorable conditions for male teachers’ professional development through institutional design and practical support. Specifically, while enhancing male teachers’ professional competencies, institutions should systematically guide them in exploring effective pathways to translate gender-specific traits into practical teaching abilities. Male teachers may also be encouraged to appropriately extend beyond standardized curriculum requirements—building on foundational teaching guidelines—by assuming a more proactive role in curriculum design, development, and implementation, flexibly adopting instructional approaches and pedagogical styles that align with their personal attributes, thereby progressively strengthening their capacities in educational activity planning and execution, communication and collaboration, and child support and guidance. When male preschool teachers succeed in establishing their irreplaceable value within the early childhood education field through distinctive teaching styles and professional competence, they are more likely to earn recognition and respect from colleagues, parents, and mainstream society (Reich et al., 2021). This recognition can, to some extent, alleviate the marginalization arising from gender stereotypes. In other words, transforming gender advantages into professional competence constitutes not only a pivotal mechanism for achieving professional identity among male preschool teachers but also a long-term strategy for overcoming implicit exclusion and constructing a stable professional identity.
(2) Designing role model incentive programs. At present, most preschools face the dual challenge of a quantitative shortage of male preschool teachers and a scarcity of long-term practitioners. The lack of established male role models in the field objectively constrains the professional development horizons and growth opportunities for male teachers. Meanwhile, many preschools have yet to develop individualized training and development mechanisms tailored to male teachers, making it difficult to fully harness their gender-specific advantages and individual potential, thereby further limiting their ability to realize professional value in practice. Therefore, in supporting the development of male teachers, preschool administrators should pay close attention to their intrinsic career aspirations and developmental expectations. By designing role model incentive programs that address gender-specific characteristics, administrators can stimulate male teachers’ endogenous motivation for self-development. Specifically, administrators can identify and promote exemplary cases of male preschool teachers through multiple channels, assisting in-service male teachers with career planning and goal setting. Although the pool of male role models within a single preschool may be limited, administrators can draw upon outstanding examples of male teachers emerging nationwide, or introduce scientifically sound training models and management experience from both domestic and international contexts. Such efforts can provide male teachers with more “visible” career development pathways and growth templates, helping them clarify their future professional directions and strengthen their sense of professional belonging and developmental confidence. It is worth emphasizing that role model incentives not only help male teachers clarify their professional positioning and expand their developmental space, but also play a crucial role in their resistance to implicit social exclusion. When male teachers encounter role models who share similar circumstances yet have achieved breakthroughs in their professional trajectories, they are more likely to develop positive professional identification psychologically, alleviating the sense of isolation and anxiety stemming from gender stereotypes (Xu, 2025). The presence of such role models serves both as tangible evidence that “men can also achieve long-term development in early childhood education”, and as a form of subtle social support. This support can effectively mitigate male teachers’ feelings of powerlessness when confronting implicit exclusion, assisting them in constructing a more stable and confident professional identity through their professional practice.
(3) Expanding the space for building a professional community for male and female teachers. Positive professional relationships not only enhance workplace collaboration and foster the development of individual teachers’ professional competencies, but also effectively increase job satisfaction and professional commitment while alleviating occupation-related negative emotions (Klassen and Chiu, 2010). Therefore, to facilitate male teachers’ better integration into the early childhood education workplace, it is imperative to pay greater attention to their psychological belonging and emotional needs within the professional community, and to continuously deepen the construction of such a professional community. From the perspective of power relations, the entry of male preschool teachers has disrupted the longstanding female-dominated, single-gender occupational structure in the field of early childhood education. This means that as men attempt to integrate into the early childhood education workplace, they inevitably encounter a certain degree of tension and collision with the existing traditional power boundaries shaped by female dominance. To avoid a zero-sum game in power relations resulting from shifts in gender structure, preschool administrators should proactively cultivate a discourse system centered on gender equality. For instance, by encouraging qualified male and female teachers to fairly compete for leadership positions, administrators can continuously reinforce the legitimate connection between “professional competence” and “workplace authority”, guiding both male and female teachers to jointly maintain workplace order and gradually construct a professional community characterized by mutual complementarity and reinforcement. Within such a community, newly recruited male preschool teachers can gain a more authentic sense of professional belonging through close professional ties and group interactions, rather than becoming trapped in isolated, competing “gender alliances.” In a professional community genuinely grounded in professional identity and bound together by collaborative symbiosis, male teachers are no longer reduced to bearers of “gender labels”, but are instead accepted and recognized as colleagues possessing professional value. This form of identity, based on professional relationships, can effectively alleviate the implicit pressures stemming from mainstream gender discourse in society (Jung, 2012), helping male preschool teachers establish psychological buffers against external exclusion within the workplace.

## Concluding remarks

Based on the research paradigm of grounded theory, this study explores the paradox between “high social expectations” and “high turnover rates” in the identity construction of male preschool teachers in China from the perspective of implicit social exclusion. Following the research procedures of grounded theory, this study identified, synthesized, and constructed a theoretical model grounded in the empirical realities and professional contexts of 32 male preschool teachers, revealing the cultural exclusion, economic exclusion, and unconscious relational exclusion underlying the high turnover rate among this population.

Secular society in China indeed expects men to assume the responsibility of cultivating a traditional masculine image and to achieve success in the conventional sense. The findings indicate that as male preschool teachers age, the tension between their “playful” professional persona and the “mature and reliable” image traditionally expected by society intensifies, placing them under considerable pressure to fulfill conventional gender obligations within mainstream discourse. In their social lives, male preschool teachers, like other men, also confront the “struggle between roses and bread”. Against the backdrop of modest salaries and dim prospects for future income growth, male preschool teachers find it difficult to meet the gendered expectation of serving as “breadwinners” in Chinese society, and to demonstrate their masculinity respectably by shouldering greater economic responsibilities. Moreover, the implicit cultural and economic exclusion experienced by male preschool teachers within social networks—often manifested through “non-malicious teasing” or “unconscious bias” rooted in stereotype threat—implicitly signals that their career choices deviate from the gender norms subconsciously held by mainstream social groups. These intertwined forms of implicit social exclusion collectively constrain the construction of male preschool teachers’ subjective identity in terms of professional values, beliefs, and emotions, gradually eroding their professional commitment.

Due to limitations in the scope of observation and research perspective, this study has several shortcomings that warrant further consideration. First, although constructing a theoretical model based on the grounded theory paradigm effectively reveals the internal logic underlying the high turnover rate among male preschool teachers, as a qualitative approach, it is inherently susceptible to some degree of subjectivity or “contamination” from prior knowledge, experience, and existing theories during the process of concept and category formation. Second, although we carefully considered sample representativeness during the sampling process by selecting 32 male preschool teachers from Xi’an, Shanghai, and Nanning as interview participants, it is important to acknowledge that the level of preschool education development varies to some extent across these three cities. For instance, Shanghai’s early childhood education resources rank among the most advanced in the country, whereas Nanning’s foundation is relatively weaker. Such regional developmental disparities may lead to heterogeneity in career trajectories, thinking patterns, and professional decision-making among male preschool teachers from different areas, thereby potentially influencing the generalizability of our findings to a certain degree. Accordingly, the conclusions of this study warrant further validation and refinement through more diverse samples in future research. Finally, it should be noted that the sample in this study was limited to male preschool teachers working in public preschools. In China, public and private preschools differ substantially in terms of employment mechanisms, welfare benefits, and career development pathways. Male preschool teachers in private preschools often operate within a more competitive market-driven employment environment, lacking the job stability inherent in the public sector. Compared with their counterparts in public institutions, these institutional differences may further intensify or reshape the forms and experiences of implicit social exclusion they encounter. Consequently, this institutional divide may also constrain the external validity of our findings—that is, the mechanisms restricting identity construction revealed in this study may not fully capture the unique challenges faced by male preschool teachers in private early childhood education settings. Future research could enhance the credibility of its conclusions by expanding sample sizes and optimizing sample composition, particularly by including male teachers from private preschools in comparative analyses, thereby elucidating how institutional contexts moderate the process of identity construction.

## Data Availability

The original contributions presented in the study are included in the article/supplementary material, further inquiries can be directed to the corresponding author.
